# Complementary feeding practices and associated factors in Damot Weydie District, Welayta zone, South Ethiopia

**DOI:** 10.1186/s12889-018-5245-8

**Published:** 2018-03-27

**Authors:** Bereket Epheson, Zewdie Birhanu, Dessalegn Tamiru, Garumma Tolu Feyissa

**Affiliations:** 1 Regional Health Bureau of the Southern Nations, Nationalities and Peoples’ Region, Hawassa, Ethiopia; 2 Jimma University, Department of Health, Behaviour and Society, Jimma University Institute of Health, Jimma, Ethiopia; 3 Jimma University, Department of Population and Family Health, Jimma University Institute of Health, Jimma, Ethiopia

**Keywords:** Complementary feeding, Dietary diversity, Meal frequency, Ethiopia

## Abstract

**Background:**

Each year, more than millions of under-five children die due to under-nutrition, and many of these deaths are associated with inappropriate feeding practices. This study aimed to assess complementary feeding practices in Damot Weydie District, South Ethiopia.

**Methods:**

A community-based cross-sectional study was conducted among four-hundred and one mothers who had children aged 6–23 months in Damot Weydie District. A pretested structured questionnaire was used to collect data using a face-to-face interview. Data were entered into Epi-Data version 3.1 and analysis was done by using Statistical Package for Social Sciences (SPSS) version 20. Multivariable logistic regressions were conducted to determine independent factors associated with complementary feeding practices.

**Results:**

More than half (50.6%) of children were given complementary foods at six months of age. Only 8.5% of young children aged 6–23 months were fed with appropriate complementary foods. The proportion of mothers who reported that they know that a baby of 6–23 months old should be fed two or three times was only 75.8%. Government-employed mothers (adjusted odds ratio (AOR) = 0.14(0.04, 0.50) and mothers who attended postnatal care (AOR = 0.19(0.05, 0.70) were less likely to practice inappropriate complementary feeding. Mothers having children with birth intervals less than 35 months were more likely to practice inappropriate complementary feeding when compared to mothers of children with birth intervals greater than 35 months (AOR = 2.67 (1.22, 5.83).

**Conclusions:**

Considerable proportions of infants and young children were not appropriately fed with complementary foods as per WHO recommendations. Being a government employee mother, attending postnatal care and having a child with birth interval greater than 3 years were associated with appropriate complementary feeding. Therefore, it is important to encourage postnatal care utilization and incorporate complementary feeding advice during postnatal visits. It is critical to raise the awareness of the community about optimal complementary feeding practices with special attention to unemployed and less educated mothers. Additionally, inter-sectoral collaboration should be strengthened to increase the variety of food groups available.

## Background

According to the Ethiopian Demographic and Health Survey (DHS) data of 2016, 38% of under-five children are stunted and 10% of them are wasted [1]. Global evidence suggests that 15% deaths in under-five children can be averted if populations could access evidence-based interventions with 90% coverage [2].

The World Health Organization (WHO), at its 54th health assembly urged member states ‘to strengthen activities and develop new approaches to protect, promote and support exclusive breastfeeding for six months’ (pp.2) [3] and ‘to provide safe and appropriate complementary foods, with continued breastfeeding for up to two years of age or beyond, emphasizing channels of social dissemination of these concepts in order to lead communities to adhere to these practices (pp.3) [3].

The WHO defines complementary feeding as “the process starting when breast milk alone is no longer sufficient to meet the nutritional requirements of infants, and therefore other foods and liquids are needed, along with breast milk.” pp.8 [4]. Starting complementary feeding too soon and delaying for too long is also not advisable. The early exposure of infants to microbial pathogens potentially contaminating complementary foods and fluids puts them at increased risk of diarrheal diseases and consequently malnutrition and breast milk alone may not provide enough energy and nutrients and may lead to growth faltering and malnutrition respectively [5]. Therefore, the WHO recommends that complementary feeding should commence at six months after birth [4].

The frequency of meals required by an infant or a young child in a day depends on the amount of energy that the young infant or the child needs, the amount that a child can eat at each meal and the energy density of the food offered. Breastfed children need only non-liquid foods, whereas. Non-breastfed children should get both milk and solid or semi-solid foods [6, 7]. Appropriate feeding of children aged 6–23 months is multidimensional. Hence it is essential to have a composite indicator that tracks the extent to which these multiple dimensions of adequate child feeding are being met. The minimum acceptable diet is an indicator that combines standards of dietary diversity and feeding frequency and breastfeeding status. It helps to track progress at simultaneously improving the key quality and quantity dimensions of children’s diets [7, 8].

A complementary feeding that is provided based on the WHO recommendations promotes growth of the child and prevents stunting among children between 6 and 23 months of age [9]. However, according to the Ethiopian DHS data of 2016 only 7seven percent of children aged 6–23 months were reported to have met the minimum acceptable diet [1]. Few localized studies in Ethiopia, indicated that complementary feeding practices were inadequate [10, 11]. Still there are variations across the study findings, especially with respect to timing of initiation of complementary feeding [10–12]. Given that there are variations in feeding practices across study settings, this study aimed to assess complementary feeding practices in Damot Weydie District, which is found in Southern Nations and Nationalities People’s Representative (SNNPR).

## Methods

### Study setting and design

This community-based cross-sectional study was conducted from March to April 2014 in Damot Weydie District, which is located at 26 Km to the east of the zonal capital of Welayta Sodo and 368 Km from Addis Ababa. Damot Weydie is one of the rural districts in Welayta Zone. The district has 25 kebeles (the lowest administrative level). All the kebeles are accessible in both dry and rainy seasons. There is one main road which connects the district with zonal and regional towns. Based on projection from 2007 population and housing census report, the total population in 2013/14 was estimated to be 112,065 where 55,696 are males and 56,369 are females [13]. Most of the residents depend on traditional subsistence agriculture for living. The main crops produced in the area are maize and sorghum (Weyde District D. 2013.Health office, Annual Report[unpublished]*).*

#### Target population

The source population for the current study was all children aged 6–23 months with their mothers/care givers residing in Damot Weydie District. Those mothers and children who do not meet the inclusion criteria (children less 6 months or above 2 years of age) and mothers who were unable to respond due to some reasons were excluded from the study.

Sample size and sampling technique.

Sample size was determined by using single proportions formula with the assumptions of P (minimum meal frequency of infant and young child) of 48.9% [11] in Southerns Nations and Nationalities Peoples Representative (SNNPR) and a 95% confidence interval with margin of error 5%. With these assumptions, a sample size of four hundred and four was obtained. Eight kebeles were randomly selected from the list of 25 kebeles. The sample was then allocated for the selected kebeles by proportionally based on the population size of each kebele. Finally, study participants were selected by using simple random sampling from each selected kebele. The eligible target children were selected according to the sampling frame created based on record obtained from Health Extension Worker’s (HEW’s) family folder and from each household of eligible child aged 6–23 months and who had mother/care giver at the time of the study.

### Study variables

**Dependent variable:** The dependent variable was complementary feeding practices.

**Independent variable:** The independent variables of the study were:

- Socio-economic and demographic characteristics such as age, marital status, family size, educational status, husband educational status, occupational status, family wealth,
- Maternal health service and related characteristics such as parity, ANC visit, place of delivery, PNC visit, knowledge of mothers, water supply, source/access to information
- Child related characteristics such as age, sex, birth order and birth interval.

### Data collection tool and measurement

The structured interviewer-administered questionnaire was used to collect data. The questionnaire was developed in English, translated into Wolaita Doonaa (local language), and back translated to English to check its conceptual equivalence by a person who can speak both languages. A 24-h dietary recall method, which is the most widely used time-period for collecting dietary information, was used to collect dietary information. The quality of the data was assured through a pre-test conducted in a similar setting (Sodo Zuria). The consistency and flow of the questionnaire was checked during the pretest. Training was given for data collectors and supervisors. Completed questionnaires were cross-checked by data collection supervisors daily.

#### Data processing and analysis

Data were entered into Epi-Data 3.1 statistical software, and then exported to Statistical Package for Social Sciences (SPSS) version 20 for analysis. Descriptive analysis such as frequencies, proportions, and means were calculated. Binary logistic regressions were used to determine the strength of association between independent and dependent variables using odds ratios (OR) and 95% confidence intervals. Variables with *P* ≤ 0.25 in bivariate analysis were selected for multivariable analysis. In multivariate logistic regression analysis, both crude and adjusted odds ratios were calculated to identify independent factors associated with appropriate complementary feeding practices and *P* value ≤0.05 was considered as significant.

In this study, timely introduction of complementary feeding was defined as the proportion of children aged 6–23 months who started complementary foods at 6th month. Minimum dietary diversity was defined as the proportion of children aged 6–23 months who received foods from four or more food groups from seven food groups. The seven food groups used for tabulation of this indicator were grains, roots and tubers; legumes and nuts; dairy products (milk, yogurt, cheese); flesh foods (meat, chicken and liver/organ meats); eggs; vitamin ‘A’ rich fruits and vegetables; and other fruits and vegetables.

Minimum meal frequency was defined as the proportion of breastfed and non-breastfed children aged 6–23 months who received solid, semi-solid, or soft foods the minimum number of times or more. For breastfed children, the minimum number of times varies with age (2 times if 6–8 months and 3 times if 9–23 months). The minimum acceptable diet was defined as the proportion of breastfed children aged 6–23 months who had at least the minimum dietary diversity and the minimum meal frequency during the previous day [1].

## Results

Four-hundred and one caregivers participated in the study with a response rate of 99.3%. Majority of participants (97.8%) were married. The mean (±SD) age of the caregivers was 30.26 (±4.98) years with the range of 21–40 years. More than half of caregivers (55.1%) had no education. The mean age of children was 1.16(±0.38 years) (Table 1).

**Table 1:** Socio demographic characteristics of mothers of study participants, Damot Weydie district, Welayta zone, Ethiopia, 2014

Variables	Categories	Frequency (%)
Ethnicity	Welayta	396(98.8)
	Amhara	4(1)
	Gurage	1(0.2)
Religion	Protestant	319(79.6)
	Orthodox	73(18.2)
	Catholic	9(2.2)
Husband education	No education	179(44.6)
	Primary	124(30.9)
	Secondary +	98(24.4)
Age of mother	20–24 years	59(14.7)
	25–29 years	115(28.7)
	30–34 years	121(30.2)
	35–40 years	106(26.4)
Marital status	Single	7(1.7)
	Married	392(97.8)
	Widowed	2(0.5)
Family size	Below 5	186(46.4)
	Above 5	215(53.6)
Mother occupation	House wife	268
	Government employee	15
	Others^a^	118
Household wealth index	Poorest	87
	Poorer	73
	Middle	82
	Richer	79
	Richest	80
Place delivery	Home	180
	Health institution	221
Age of child	6–11 months	129
	12–17 months	176
	18–23 months	96
Sex of child	Male	207
	Female	194

^a^Students, Private business, Farmer

**Fig. 1:**
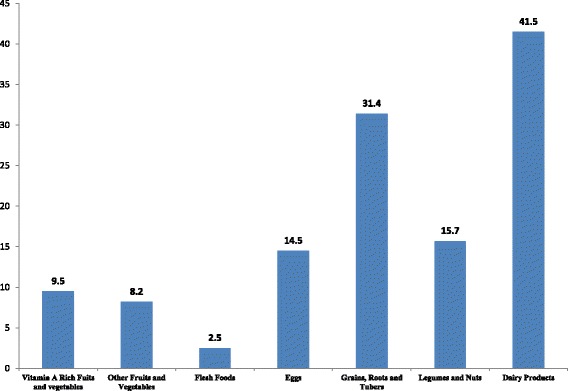
Types of foods given to children aged 6–23 months in Damot Weydie district, Welayta zone, SNNPRS, 2014

The current study showed that majority (97%) of mothers had heard about complementary feeding practices and 165 (41.15%) knew about the harmful effect of bottle-feeding. The mean knowledge score about complementary feeding of study participants was 7.95 (SD = 1.327) (Table 2). One hundred and twenty-six (31.4%) mothers provided food made from grains while 177(44.1%) of them gave dairy products for their children. Small number of caregivers (15.7%) fed their children in the previous day. Only 38(9.5%) of children ate foods that contained Vitamin A rich fruits and vegetables during the day or night. Overall, half of the children (50.6%) aged 6–23 months consumed some solid or semi-solid foods during the day or night (Fig. 1).

**Table 2:** Caregivers’ knowledge of complementary feeding practices at Damot Weydie district, Welayta zone, SNNPRS, 2014

Knowledge items	Frequency (%)
Heard about complementary feeding	389(97)
Heard when to start complementary food	388(96.8)
Know what types of foods to feed young children	384(95.8)
Know how to feed young children	376(93.8)
Know when to start complementary food	280(69.8)
Know baby of 6–23 months should eat 2/3 times	304(75.8)
Know feeding anything from a bottle not good	165(41.1)
Know thicker porridge do not chock the child	328(81.8)
Know hand washing before food preparation	387(96.5)
Know hand washing with soap/ash after toilet	369(92)

**Fig. 2:**
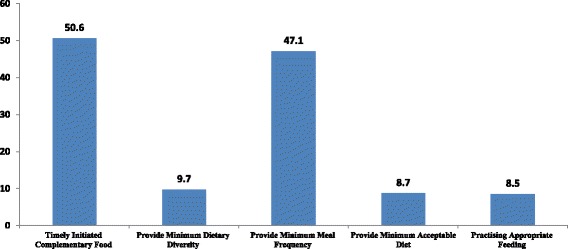
Proportion of complementary feeding practices of infant and young children (6–23 months) in Damot Weydie District, Welayta zone, SNNPRS, 2014

Our study showed that only 8.5% of young children aged 6–23 months were fed with appropriate complementary foods. More than half (50.6%) of mothers introduced complementary foods at 6 months of age as per WHO recommendation. Nearly, 396(98.8%) children received breast milk during the 24-h period and 189(47.1%) were fed at least the minimum number of times. In total, 34 (8.5%) of the children were appropriately fed with complementary foods (Fig. 2).

In the bivariate analysis, postnatal care, occupational status of the mother, childbirth interval and hearing about complementary feeding from health workers were found to be associated with complementary feeding practices (data not shown). In multivariate logistic regression analysis, mother’s occupation, preceding birth interval of the child and postnatal care were independent factors associated with appropriate complementary feeding practices. Mothers who were government employees were 85.1% less likely to practice inappropriate complementary feeding when compared to those of housewives (AOR = 0.14 (0.04, 0.50). Children born with less than 35 months (< 3 years) preceding birth intervals were 2.67 times more likely to be fed with inappropriate complementary feeding when compared to children born with greater than 35 months (< 3 years) birth intervals (AOR = 2.67 (1.22, 5.83). Mothers who attended postnatal care were 80.2% less likely to practice inappropriate complementary feeding compared to mothers who did not attend (AOR = 0.19(0.05, 0.70) (Table 3).

**Table 3:** Complementary feeding practices and associated factors in Damot Weydie district, Welayta zone, SNNPRS, 2014

Characteristics		Complementary Feeding Practices			
		Appropriate	Inappropriate	COR (95% CI)	AOR (95% CI)
Mother’s occupation	House wife	7.5	92.5	1	1
	Government employee	33.3	66.7	0.16(0.05, 0.52)	0.14(0.04, 0.50) ++
	Others^a^	7.6	92.4	0.97(0.43, 2.21)	0.72(0.31, 1.70)
Preceding Birth interval	< 35 months	6.8	93.2	2.29(1.09, 4.78)	2.67(1.22, 5.83) +
	35^*+*^ months	14.3	85.7	1	1
Attending PNC	Yes	10.4)	89.6	0.25(0.07, 0.85)	0.19(0.05, 0.70) +
	No	2.9	97.1	1	1
*Sex of the child*	Male	10.1	89.9	0.63(0.30, 1.30)	0.50(0.23, 1.07)
	Female	6.7	93.3	1	1

^a^Farmer, Student, Private Business; ++Significant at *P* < 0.01, +Significant at *P* < 0.05

*COR* Crude odds ratio, *AOR* Adjusted odds ratio, *CI* Confidence Interval

## Discussion

The results of this study revealed that complementary feeding was timely introduced to 50.6% of infants and young children included in this study. This finding is in agreement with the findings of the study conducted in Eastern Ethiopia, which reported a prevalence of 54% [10]. However, it is slightly lower when compared to the finding of a previous study conducted in Northern Ethiopia, which reported a prevalence of 62.8% [12]. In addition, our finding was lower when compared to the findings from other countries like United Arab Emirates (83.5%) [9], Tanzania (92.3%) [14], coastal South India (77.5%) [15] and Nepal (70%) [16]. Among children aged 6–23 months, 9.7% received foods from at least four food groups or more (minimum dietary diversity). It was higher than the regional state data of the Southern Nations and Nationalities People’s Representatives (SNNPR) regional state which was 2.5%. The relatively higher proportion might be due to practice change with time, and the contribution of community-based nutrition interventions led by health extension workers.

About 47.1% of infants and young children (6–23 months) were fed at least with the minimum required meal frequency. This finding is low when compared to findings from Nepal, which was 82% [16]. The difference might be due to low maternal educational status about optimal child feeding practices in our study area. The proportion of mothers who knew that a baby of 6–23 months old should be fed two or three times was only 75.8%. This finding underscore that it is essential to raise the awareness of the community regarding the optimal frequency of complementary feeding.

The minimum acceptable diet (either four or more food groups and minimum meal frequency) reported in this study (8.7%) was relatively higher when compared to findings of other studies from southern Ethiopia [12]. This might be due to the recent initiation of inter-sectoral collaboration of nutrition programs. However, it is comparable to the 2016 national level data of Ethiopia (7%) [1].

Finding of this study showed that a small proportion of caregivers (8.5%) were practicing appropriate complementary feeding which was lower than findings from Zambia (25.1%) [17]. This might be due to poor socioeconomic status and lower maternal literacy, or due to unavailability of the food varieties, which may make mothers incapable of fulfilling dietary diversity and frequency of meal.

Government-employed mothers were less likely to practice inappropriate complementary feeding when compared to those mothers who were housewives, which is similar with findings of the study from Nepal [16]. This might be due to the fact that women’s employment increases household income and access to health information with consequent benefit to household nutrition in general and child feeding practices in particular [18–20]. This study also showed that higher birth intervals preceding the child (more than 3 years) were significantly associated with appropriate feeding practices, which is in agreement with the findings from Nepal and other developing countries [16, 18, 19]. This might be due to availability of sufficient time to give care for a child before giving other birth.

Our findings also indicated that caregivers who attended postnatal care were more likely to practice appropriate complementary feeding when compared to those care givers who did not attend postnatal care. This result was in congruent with the findings from Northwestern Tigray, Tanzania, India and Nepal [14–16, 21]. This might be due to the result of counseling that the mothers receive from health professionals during their postnatal visits. However, this study showed that there was no statistically significant association between maternal education and appropriate complementary feeding practice and it disagrees with the study reports from Nepal and India [15, 16].

Even though this study addressed complementary feeding practice in Damot District, it should be interpreted with the following limitations. Child feeding practices were age-specific with narrow age ranges and typically assessed by caregivers’ report or recall which can lead to recall bias. Seasonal variations might affect the food groups consumed by the infants and young children during the time of the interview. The cross-sectional nature of the study itself poses difficulty in establishing cause-effect relationship.

## Conclusions

Significant proportions of children were not appropriately fed with complementary foods as per the WHO recommendations. Being a government employee mother, attending postnatal care and a higher (> 3 years) birth interval preceding the infant and young child were associated with appropriate complementary feeding. This implies that unemployed mothers and mothers who did not attend postnatal care were less likely to adhere to complementary feeding practices as per the WHO recommendations. Therefore, it is important to encourage postnatal care utilization and incorporate complementary feeding advice during postnatal visits. In addition, unemployed and less educated mothers should be given special attention in teaching about complementary feeding practices. The proportion of mothers who reported that they know that a baby of 6–23 months old should be fed two or three times was only 75.8%. Therefore, it is essential to raise the awareness of the community regarding the optimal complementary feeding practices, with special attention on frequency of feeding. Additionally, inter-sectoral collaboration should be strengthened to increase the variety of food groups.

## Abbreviations

ANC: Antenatal Care
AOR: Adjusted Odds Ratio
COR: Crude Odds Ratio
EDHS: Ethiopian Demographic and Health Survey
SNNPRS: South Nations Nationalities and Peoples Regional State
SPPSS: Statistical Package for Social Sciences
